# One Dimensional ZnO Nanostructures: Growth and Chemical Sensing Performances

**DOI:** 10.3390/nano10101940

**Published:** 2020-09-29

**Authors:** Abderrahim Moumen, Navpreet Kaur, Nicola Poli, Dario Zappa, Elisabetta Comini

**Affiliations:** Sensor Lab, Department of Information Engineering, University of Brescia, 25123 Brescia, Italy; a.moumen@unibs.it (A.M.); n.kaur001@unibs.it (N.K.); nicola.poli@unibs.it (N.P.); dario.zappa@unibs.it (D.Z.)

**Keywords:** 1D nanostructures, ZnO, VLS, catalysts, nanowires, conductometric sensors, response, selectivity

## Abstract

Recently, one-dimensional (1D) nanostructures have attracted the scientific community attention as sensitive materials for conductometric chemical sensors. However, finding facile and low-cost techniques for their production, controlling the morphology and the aspect ratio of these nanostructures is still challenging. In this study, we report the vapor-liquid-solid (VLS) synthesis of one dimensional (1D) zinc oxide (ZnO) nanorods (NRs) and nanowires (NWs) by using different metal catalysts and their impact on the performances of conductometric chemical sensors. In VLS mechanism, catalysts are of great interest due to their role in the nucleation and the crystallization of 1D nanostructures. Here, Au, Pt, Ag and Cu nanoparticles (NPs) were used to grow 1D ZnO. Depending on catalyst nature, different morphology, geometry, size and nanowires/nanorods abundance were established. The mechanism leading to the VLS growth of 1D ZnO nanostructures and the transition from nanorods to nanowires have been interpreted. The formation of ZnO crystals exhibiting a hexagonal crystal structure was confirmed by X-ray diffraction (XRD) and ZnO composition was identified using transmission electron microscopy (TEM) mapping. The chemical sensing characteristics showed that 1D ZnO has good and fast response, good stability and selectivity. ZnO (Au) showed the best performances towards hydrogen (H_2_). At the optimal working temperature of 350 °C, the measured response towards 500 ppm of H_2_ was 300 for ZnO NWs and 50 for ZnO NRs. Moreover, a good selectivity to hydrogen was demonstrated over CO, acetone and ethanol.

## 1. Introduction

Nowadays, domestic or industrial accidents caused by dangerous chemical compounds and pollutants demonstrate the real need for early detection systems. As a result, these detection devices have many potential applications in significant fields such as transportation, environment, health, industry and agriculture [1,2,3,4]. Among different types of chemical sensors, conductometric sensors based on metal oxides (MOXs) materials have several advantages, such as easy integration (compatibility) with current electronics, low production cost and suitability for a potential miniaturization. Nonetheless, these sensors still have some limitations such as selectivity and sensitivity at low working temperatures [5]. To overcome these problems, various studies have been performed to get the optimal morphology and crystalline structure. Many of them improved the detection capability, either by optimizing the synthesis method by modifying the surface properties, decorating with other materials, adding dopants or manufacturing p-n junctions to improve sensitivity to target gases. An example of the latter is reported by Kaur et al. who have enhanced the NiO sensitivity and selectivity by synthesizing a NiO/ZnO heterojunction [6,7,8]. Moreover, hybrid carbon—metal oxide heterojunctions such as graphene oxide-ZnO and graphene oxide-SnO_2_—have shown capability for selective room-temperature detection of low concentration volatile organic compounds [9,10]. Moreover, a new approach was provided by Hu-Jun Le et al. for designing versatile hydrogen sensors using alloy@oxide core-shell, such as PdAu_alloy_@ZnO core-shell, as sensing material with high response and excellent selectivity to hydrogen [11].

The rapid development of nanoscience and nanotechnology has greatly pushed the scientific community and industrial companies to explore new features of both conventional and novel materials at the nanoscale level. Thanks to nanotechnology, it becomes possible to develop materials by controlling their structure at atomic level, resulting in new properties for the material in order to be used in all applications fields and chemical sensors in particular. Recently, one-dimensional (1D) metal oxide nanostructures, such as nanowires and nanorods, are addressing this challenge, attracting new possibilities and offering considerable characteristics for the fabrication of various nanodevices. The controlled synthesis of one-dimensional nanomaterials, finding new strategies to enhance the performance of their practical applications such as chemical sensors, is a hot topic for the scientific committee in materials science field. The high crystallinity, density of states, 1D charge transport and high specific area are key features for the next generation of nano-conductometric sensors in near future [12].

Zinc oxide is a n-type semiconductor with a narrow band gap energy around 3.3 eV, high thermal and chemical stability, large exciton binding energy of 60 meV at room temperature, high electron mobility, non-toxic material and environmentally friendly. Those characteristics make ZnO a strong concurrent to substitute expensive materials such as In_2_O_3_ and WO_3_ in many applications.

The present work reports several deposition conditions and characteristics that allow the precise control over the shape, density, form and orientation of 1D ZnO nanostructures. For this purpose, vapor-liquid-solid (VLS) mechanism was selected, characterized by its simplicity, low cost, reproducibility and feasibility for the deposition of high quality 1D nanostructures over a large area [13]. The material to be deposited is evaporated, transported and condensed on top of catalyst clusters on substrates [14]. In particular, catalysts may be used to control the aspect ratio, shape and morphology, as reported previously in literature. Yang et al. studied the orientation, positional control, density and diameter of ZnO by dispersing Au clusters using different thin films thickness. A direct relation was extracted confirming the dependence of Au nanoparticles (NPs) size with nanowire’s diameter [15]. Yanagida et al. interpreted the effect of catalyst size on the adsorption and diffusion of surrounding atoms that comprises MgO vapor. A systematic study was performed, confirming that the diffusion ratio of atoms within the catalyst droplet decreased by increasing catalyst size [16]. On the other hand, Zappa et al. studied the effect of Pd, Au and Sn catalysts on SnO_2_ nanowires growth with VLS using different metals while keeping the growth temperature and the other experimental conditions constant. A surprising result was found: no morphology transition was observed and similar SnO_2_ nanowires were produced with the use of all catalyst, which means that catalyst nature did not have a huge impact on supersaturation and crystallization of nanowires [17]. Unfortunately, most of these studies lack an investigation of the catalyst’s effect on the nanostructure’s functional performances. In this study, the impact of catalyst nature on the structural, morphological and electrical properties of 1D ZnO nanostructures are studied, and the sensing properties towards reducing gases are evaluated in order to fabricate high performance and low cost conductometric sensors based on 1D ZnO.

The present work provides a new approach linking vapor liquid solid (VLS) mechanism with enhancing sensing performance. In fact, the double role of the metal catalyst is evidenced, namely controlling the growth mechanism of 1D ZnO and leading to superior sensing performance due to chemical (spillover) or electronic sensitization or even promoting bulk doping.

## 2. Materials and Methods

### 2.1. Material Preparation

Alumina (99.9% purity, 2 mm × 2 mm, Kyocera, Kyoto, Japan) and silicon substrates were used as a support for growing 1D ZnO nanostructures. Alumina was used for the fabrication of chemical sensors, whereas silicon was used for material characterization. Prior the deposition, samples were ultrasonically cleaned in acetone (Carlo Erba, Milano, Italy) solvent for 15 min and then dried in synthetic air to remove any dust or contamination. Magnetron sputtering was used to catalyze the substrates, coating them with ultrathin films of gold, platinum, silver and copper (Au, Pt, Ag, Cu) with thickness values between 2 and 7 nm, according to the experimental conditions reported in Table 1. To the best of our knowledge, the present work is the first study reporting the use of Cu as a catalyst for the synthesis of ZnO nanowires. Instead, Ag was used in few previous works [18,19], but the achieved NWs were not exploited for chemical sensing applications. In VLS growth mechanism, catalysts play a major role and behave as nucleation sites for ZnO growth, controlling the diameters of the nanostructures and moreover may enhance the sensing performances.

To synthetize the nanostructures, ZnO powder (99.9% purity, Sigma Aldrich, St. Louis, MO, USA) was placed in the middle of alumina tube and heated at 1200 °C, keeping the pressure at 10 mbar to induce the powder evaporation. The ZnO vapor is then transported to a colder region of the furnace using a carrier gas flow (argon, 75 sccm) where it condenses on the substrates covered by catalysts. The deposition process was kept for 15 min. Each catalyst used for the 1D ZnO growth is active at a specific temperature, i.e., Au (500 and 600 °C) Pt (600 °C), Ag (350 °C) and Cu (400 °C), confirming the temperature role in supersaturation and growth of 1D ZnO nanostructures.

### 2.2. Characterization

The structural properties of ZnO samples were extracted using X-Ray diffractometer (Empyrean; PANalytical, Almelo, The Netherland) with Cu-LFF (λ = 1.54 Å) tube operated at 40 kV–40 mA. Morphological analysis was investigated using field scanning electron microscopy (LEO 1525 model; Carl Zeiss AG, Oberkochen, Germany) operated at 10 KV and transmission electron microscopy (TEM) 200CX (JEOL, Tokyo, Japan).

### 2.3. Device Fabrication

**Figure 1:**
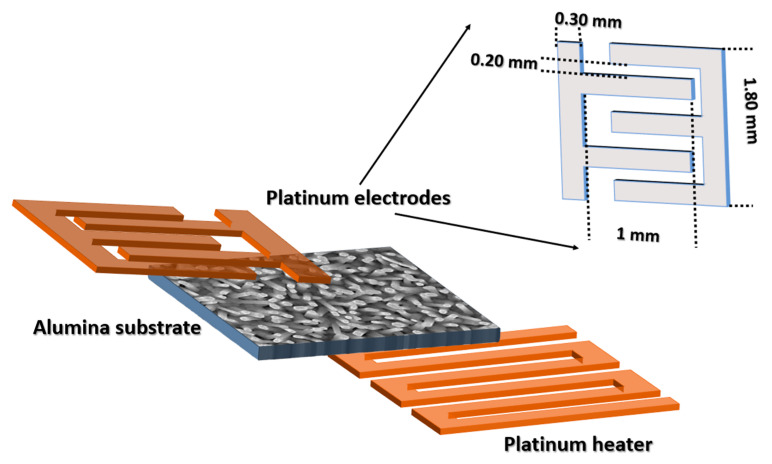
Design of conductometric sensor device.

Conductometric chemical sensors have been prepared by depositing a heater and electrical contacts on the substrates with the sensing material (ZnO), as shown in Figure 1. Firstly, TiW/Pt adhesion layer was deposited by means of DC magnetron sputtering (KENOSYSTEC SRL, Milano, Italy) (70 W argon plasma, 7 SCCM argon flow, 5 × 10^−3^ mbar pressure). Afterwards, platinum electrodes with interdigital geometry were deposited using the same conditions. Because metal oxides are generally thermally activated, DC magnetron sputtering was used to deposit a platinum heater on backside of alumina substrates (70 W argon plasma, 5 × 10^−3^ mbar pressure). Finally, alumina substrates were mounted on transistor outline (TO5) packages using electro-soldered gold wires [20,21].

The gas sensing tests were performed inside a stainless-steel chamber at the fixed temperature of 20 °C. Several gases such as hydrogen, CO, ethanol and acetone were tested at 50% relative humidity. The concentration (in ppm) of each gas was achieved by mixing the gas with the synthetic air. The flow was fixed at 200 sccm and the electrical conductance was measured using picoammeter (Keithley, Solon, OH USA) by applying 1 V. The desired gas concentration has been injected for 30 min and then the synthetic air was injected for 1 h to restore the baseline of the electrical conductance. The samples were tested at different working temperature ranging from 200 to 500 °C. Three different samples were prepared for each catalyst in the same conditions. The presented results are the average of the measurements performed on all devices. The response of the ZnO sensor towards reducing gases was defined as $\frac{G_{gas}-G_{air}}{G_{air}}$, where *G_air_* and *G_gas_* are the sensor conductance in the absence and presence of the reducing gas, respectively.

## 3. Results

### 3.1. Surface Morphological Analysis

In this work, the control over the form, shape and morphology of 1D ZnO nanostructure was explained by two mechanisms: catalyst’s composition and thermodynamic conditions. Each catalyst is active for nanostructure formation at specific temperature, depending on its specific melting point. Liquid droplets formation and supersaturation are crucial parameters for controlling the nanostructures morphology and explaining the different shapes obtained. In general, the mechanism leading the formation of 1D ZnO structures is based on the nucleation, diffusion and crystallization phenomena [22]. The temperature plays a crucial role in VLS mechanism. Firstly, atoms in the ZnO vapor adsorb on the substrate surface. The substrates were heated from 350 to 600 °C during 15 min deposition, increasing the energy of these atoms and thus enhancing their ability to diffuse onto the substrate surface. Due to the treatment at high temperature, the catalyst particles aggregates forming liquid clusters and promoting the condensation of ZnO vapor. The ZnO vapor molecules reach the droplet catalyst surface and are incorporated as adatoms. The adatoms diffuse into the cluster and, as the supersaturation of the droplet occurs, the segregation will start forming 1D nanostructures.

**Figure 2:**
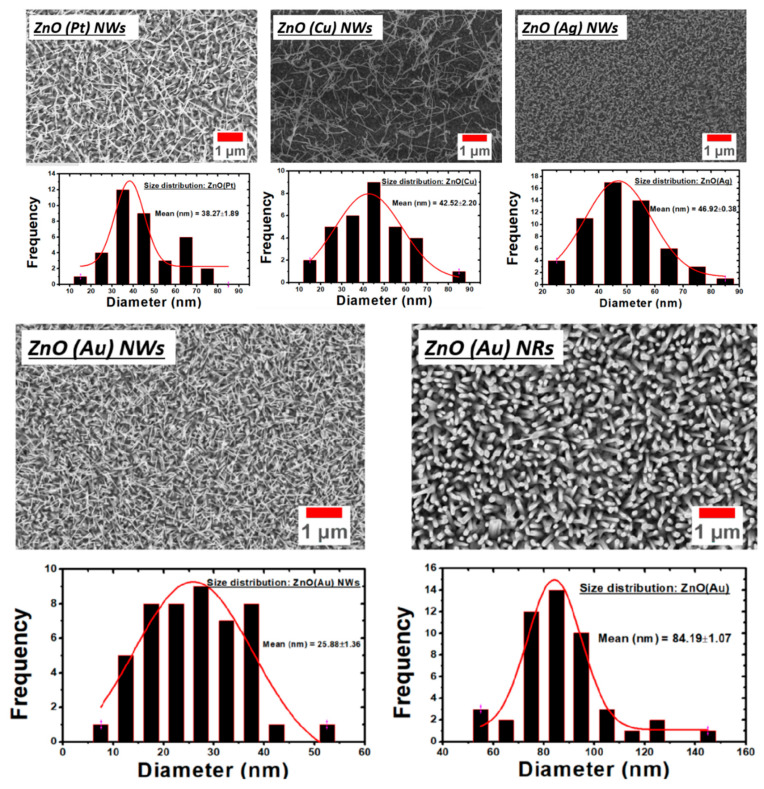
1D ZnO nanostructures using Au, Pt, Ag and Cu catalysts and its size distribution.

**Figure 3:**
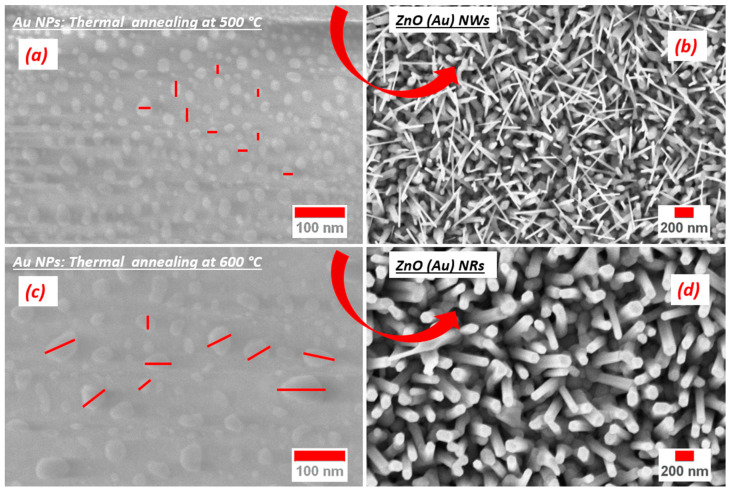
Au seed layer annealed at 500 (**a**) and 600 °C (**c**) without nano- (wire/rod) growth; High magnification ZnO nanowires (**b**) and ZnO nanorods (**d**) after growth.

Figure 2 shows ZnO nanostructures grown using Au, Pt, Ag and Cu catalysts and their respective size distribution. While the length of all samples is tabulated in Table 2. The NWs obtained using Ag catalysts are dense and homogeneous, covering completely the alumina substrate with an average diameter around 46 nm with a 342 nm length. ZnO NWs obtained using Cu catalyst are quite different in morphology: a kind of ductility of the NWs was observed, with low density. Despite that, on average they are 4 micrometres long and 42 nm in diameter. A good density of small nanowires (≈38 nm diameter and ≈842 nm length) was achieved with Pt catalyst. ZnO NWs were obtained with Au catalyst at low temperatures with a high aspect ratio and density (≈25 nm diameter and ≈771 nm length). On the other hand, high temperature promotes nanorods formation, which may be explained by the high diffusion of ZnO vapor affecting the supersaturation of ZnO and leading to the formation of ZnO NRs with high quality. The catalyst NPs size controls the diameter of crystallized NRs (≈84 nm diameter and ≈442 nm length). The morphology transition from NWs to NRs (Figure 3b,d) can be also explained by coalescence effect of the catalyst seeds. The final products consist of NPs with different size as shown in Figure 3a,c.

### 3.2. Structural Properties

**Figure 4:**
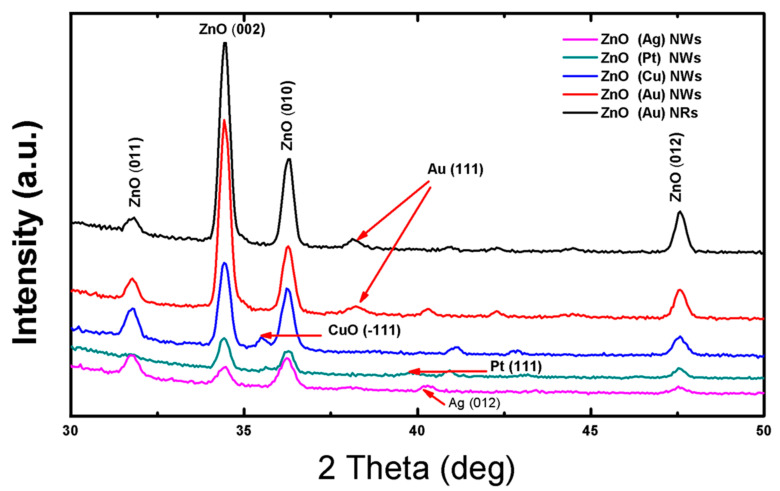
*X*-ray diffraction patterns of 1-D ZnO synthetized using various catalysts.

Figure 4 depicts the *X*-ray diffraction patterns of 1D ZnO synthetized using various catalysts. All peaks observed for the different ZnO nanostructures agree with standard ZnO (JCPDS 80-1916). The (011) (002) (010) and (012) peaks situated at 31,7°, 34.4°, 36.3° and 47.5°, respectively, show the formation of ZnO crystal belonging to hexagonal structure. The catalyst’s particles appear in their metallic or oxidized form, depending on the material. The appearance of gold (JCPDS no. b96-901-1614), platinum (JCPDS no. 98-002-1997) and silver (JCPDS no. 98-002-1958) in metallic form is confirmed by their peaks situated at 38.24°, 39.84° and 40.17°, respectively. They remain stable during the deposition even under pressure and high temperature. On the contrary, copper was oxidized into CuO (JCPDS no. 94-003-8482), as demonstrated by the peak situated at 35.56°. This is expected, considering the low chemical stability of copper. Similar results were observed by Zhang et al. [18], who studied the stability of catalyst nanoparticles before and after NWs growth. They confirmed the chemical stability of platinum and gold, while they observed and discussed the Ag oxidation to AgO, and the formation of Ag_4_SiO_4_ in case of silicon substrate. The crystallite size (D) was calculated (Table 2) using Debye Scherrer formula defined as (D = (0.94λ)/(FWHM Cosθ)), where FWHM is the full width at half-maximum of an (*hkl*) peak at θ value, θ is the half of the scattering angle and λ is *X*-Ray wavelength equals 0.154 nm. The crystallite size is 22 nm for ZnO (Au) NWs and NRs, which are smaller than the crystallite size of ZnO (Cu) NWs (D = 24 nm) ZnO (Pt) NWs (D = 30 nm) and ZnO (Ag) NWs (D = 31 nm).

The catalysts affect not only the shape and the morphology of the nanostructure, but also play a major role on the preferred orientation of crystallites. In this context, the texture coefficients of ZnO nanostructures were calculated using the equation below.

(1) $$TC\left(hkl\right)=\frac{I\left(hkl\right)}{I_{0}\left(hkl\right)}(\frac{1}{n}\sum_{i=1}^{n}\frac{I\left(hkl\right)}{I_{0}\left(hkl\right)})$$

where (*hkl*) are Miller indices denotes the *X*-ray diffraction direction plan, *I*(*hkl*) is the intensity of CuO measured, $I_{0}$(*hkl*) is the standard intensity taken from the (JCPDS 80-1916) and n is reflection number. Using the texture coefficient, the preferred orientation of crystallites could be established. The texture coefficient values of 1D ZnO nanostructures using different catalysts are tabulated in Table 3. The diffraction peak (*hkl*) with *TC* that comprises values between zero and one defines a lack in crystallites orientation *hkl*. While, when the *TC* value exceeds one (*TC* > 1), there is a majority of crystallites orientation in (*hkl*) direction. As shown in Table 3, ZnO (Au) nanowires and nanorods, ZnO (Pt) nanowires and ZnO (Cu) nanowires polycrystalline structure have (002) as preferred orientation. It is clear that ZnO (Au) NWs samples possess the highest *TC* (2.18). These results show that the diffraction peak (002) is not only the most intense peak, but also the crystallites preferential direction. As a result, (002) is the predominant orientation. On the contrary, in ZnO (Ag) nanowires (010) and (002) are both considered as preferential direction of due to their almost identical *TC* values.

### 3.3. Gas-Sensing Performace

#### 3.3.1. Working Principle

**Figure 5:**
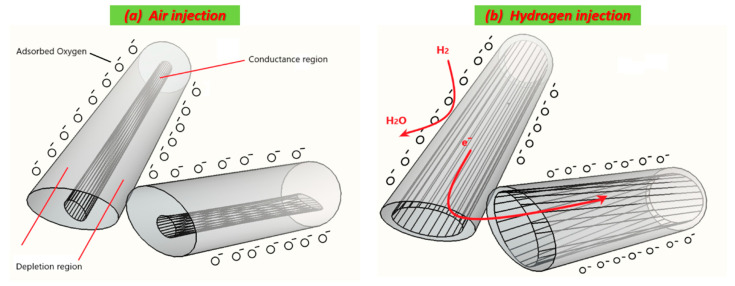
Hydrogen-sensing mechanism model of one -dimensional (1-D) sensor: (**a**) charge transfer and air injection; (**b**) sensing model toward hydrogen.

Despite the simplicity of conductometric sensors design, the gas detection mechanism remains complex and not fully understood. The gas/MOXs surface reactions, that transduce the chemical signal into an electrical one, are shortly described hereby. Oxygen is the main reaction precursor involved in conductometric sensors. As air interacts with ZnO (Figure 5a), oxygen atoms adsorb onto its surface [23]. Oxygen adsorption in oxide semiconductor materials involves a carrier charge exchange. Oxygen starts with physical adsorption at low temperatures and ends with ionic adsorption (chemisorption), yielding in the final step an anion oxygen (O^−^) at high temperatures as explained by Equations (2)–(4) [12,24]. The interaction with oxygen molecules (in air) leads to a change of the electrical conductance caused by electrons transfer from the semiconductor to oxygen ionosorbed due to its high electronic affinity.

O_2_ (gas) → O_2_ (ads) (2)

O_2_ (ads) + e^−^ → O_2_^−^ (ads) (3)

O_2_^−^ (ads) + e^−^ → 2O^−^ (ads) (4)

During gas injection, various possible scenarios may occur, depending on the semiconducting—behavior of the material (n- or p-), the injected gases nature (reducing or oxidizing), electronic affinity, ionization energy and others. When a target gas is injected, electrons exchange according to different processes. In the present work, H_2_ and the other tested gases are reducing compounds. As shown in Figure 5b, hydrogen molecules react with the adsorbed oxygen to form water molecules, as described by the Equations (5) and (6) [25]. Thanks to their small ionization energy, reducing gases behave as electrons-donors. The released electrons generated by the reaction between the reducing gases and the adsorbed oxygen cause a decrease of the thickness of the depletion region (region free of electrons) at the surface of ZnO. As a result the electrical conductance of the sensor increases [12].

(5) $$H_{2}+O^{-}\to H_{2}O+e^{-}$$

(6) $$2H_{2}+{O_{2}}^{-}\to 2H_{2}O+e^{-}$$

Moreover, in specific thermodynamic conditions, hydrogen as a reducing gas is sometimes able to remove oxygen from the ZnO crystal, creating oxygen vacancies that play a crucial role in gas sensing mechanism (Equation (7)) [26]. However, a fraction of oxygen vacancies formed is able to ionize and release one or two electrons, enhancing the electrical conduction, as explained by Equations (8) and (9).

(7) $$H_{2}+O_{O}\to H_{2}O+V_{O}$$

(8) $$V_{O}\to V_{O}^{+}+e^{-}$$

(9) $$V_{O}\to V_{O}^{++}+2e^{-}$$

#### 3.3.2. Catalyst Effect on Sensing Characteristics

**Figure 6:**
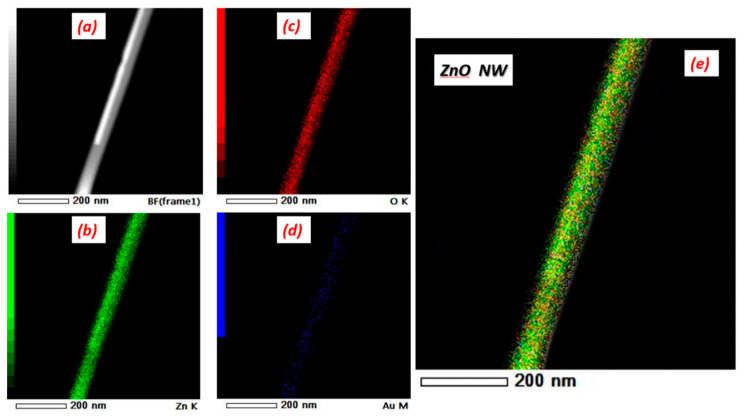
(**a**) shows ZnO transmission electron microscopy (TEM) nanowire image. TEM-energy dispersive x-ray spectroscopy (EDS) elemental mapping image of (**b**) Zn, (**c**) O, (**d**) Au and (**e**) represents the EDS mapping of single ZnO nanowire.

**Figure 7:**
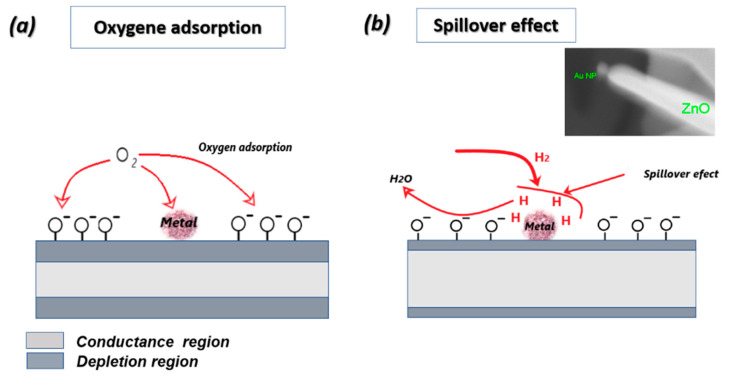
Schematic diagram exemplifying the chemical sensitization mechanism for ZnO/metal under (**a**) Oxygen; (**b**) H_2_ gas.

Despites the high quality of 1D nanostructures produced by VLS, there is a lack of understanding of the catalyst’s role especially on the functional properties. Researchers are trying to explain how the catalyst’s presence controls the VLS growth, making hypothesis about its distribution along NWs/NRs during VLS process. It may act as a doping agent, as reported by Chen et al. who have considered the catalysts as additives or contamination explaining its substitution within the crystal [27]. These catalyst’s particles may be distributed around/decorating the NWs sidewalls, as reported by Hannon et al. who have explained in detail the migration (by diffusion) of the catalyst particles from droplets to the sidewalls until the NWs growth is completely terminated [28]. In all cases, the catalyst has a crucial role in enhancing conductometric sensors characteristics. In the present work, we have analyzed all the possibilities, thanks to TEM mapping of ZnO grown by gold catalysts illustrated in Figure 6 and SEM image in the inset of Figure 7b. Au is decorating ZnO and the sensing response can be enhanced based on chemical sensitization as described in Figure 7. Gold distribution around the NWs improves gas sensing via spillover effect, by dissociating gas molecules and activating the chemical reaction on the MOX surface [29]. Gold, and in general noble metals, provides active sites for chemical adsorption, such as O_2_ adsorption (Figure 7a). At the same time, H_2_ is dissociated into fragments (atomic H) at specific temperature, and spills over ZnO surface to interact with pre-adsorbed oxygen, creating H_2_O and therefore releasing electron back to ZnO, affecting its conductance (Figure 7b). Most important, spillover effect occurs without transferring electrons from gold to ZnO. Same principle may occur for Pt and Ag, even if the latter may oxidize during the gas testing at higher temperatures. Cu interaction with the gas phase, on the contrary, may described by electronic sensitization due to the formation of stable oxide CuO. More discussions about electronic sensitization are found in literature [30]. Moreover, spillover effect does not change the gas sensing mechanism, enhancing only the rate of the chemical interaction processes.

On the other hand, many reports investigated the metallic cluster decoration which can be highly beneficial for chemical sensors applications. Moreover, bimetallic clusters have been found to be even more effective than monometallic clusters. In this context, Bahariqushchi et al. investigated free carrier enhanced depletion in ZnO nanorods decorated with bimetallic AuPt nanoclusters [31]. In comparison to ZnO NRs, the mono- and bi-metallic decorated ZnO showed high sensitivity due to increase of free carriers depletion. Furthermore, the bimetallic effect leads to an enhancement of gas adsorption and causes a stronger electron spillover from the ZnO surface to the bimetallic nanoclusters. Chen et al. investigated Au/Pd-NPs decorated ZnO nanowires for NO_2_ sensor [32]. Indeed, the enhanced sensing performance towards NO_2_ is attributed to the oxygen vacancies that have been increased in Au/Pd@ZnO sample as well as the chemical sensitization that provides more active sites for NO_2_ adsorption. Furthermore, the effect of metal decoration is well achieved also in other applications such as photocatalysts [33].

As mentioned before, the incorporation of gold into nanowires cannot be excluded, especially considering the solubility of Au in ZnO that may be achieved in specific conditions such as deposition at high pressure and growth temperature, annealing at high temperature and gold content [34,35]. However, the solubility and the chemical state of ZnO (Au) are still not clear according to the few reports in literature [35].

In some cases, it could form a ternary alloy if additive exceeds specific concentration (not expected in the present work). Instead, at low concentrations of additives, it may be a substitute in the host materials, affecting the charge carrier transfer while keeping the material the same. The impact on sensing properties is justified by the Fermi level shift due to the existence of deep donor levels within ZnO band gap energy, which can enhance the density of ionized oxygen in ZnO surface, reinforcing the reaction with reducing gases and affecting the charge depletion layer.

#### 3.3.3. Sensing Properties

**Figure 8:**
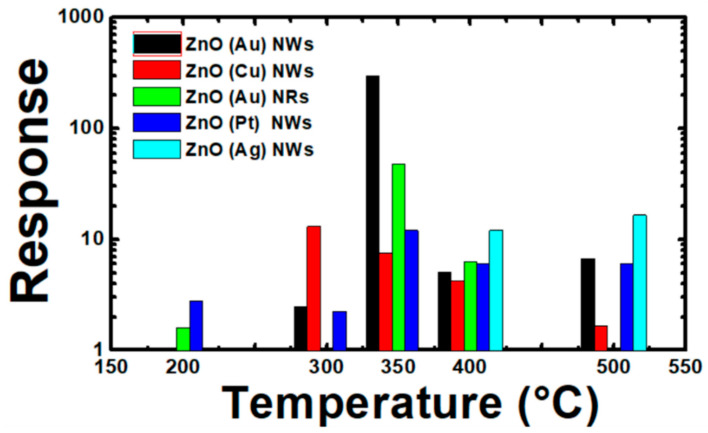
Sensors response under hydrogen injection (500 ppm) of 1D ZnO nanostructures using different catalysts as a function of temperature. The relative humidity was 50%.

In the present work, H_2_ sensors based on 1D ZnO were investigated with particular attention to the sensing characteristics such as gas response, sensors kinetic, stability and selectivity. Figure 8 shows the response of ZnO nanostructures synthetized using different catalysts towards H_2_ at several working temperatures: 200, 300, 350, 400 and 500 °C. For each ZnO sensor corresponding to specific catalyst, there is a precise temperature corresponding to the optimum response. High response was observed for H_2_ at 350 °C for all samples, but ZnO (Au) nanowires and nanorods showed the best one. However, ZnO (Cu) and ZnO (Pt) showed higher response at 300 and 200 °C, respectively. ZnO (Ag), instead, gives an appreciable response to H_2_ also at elevated temperatures (400 and 500 °C). The high sensitivity of ZnO towards H_2_ was reported by Akash Katoch et al. who proposed a sensing mechanism that considers the surface metallization of ZnO to Zn in the presence of H_2_ [36]. Indeed, the progressive ZnO to Zn transition at the surfaces of ZnO enhances electrons transport from the surface of metallic Zn to ZnO. This process affects the electrical conductance and improve the sensing properties.

**Figure 9:**
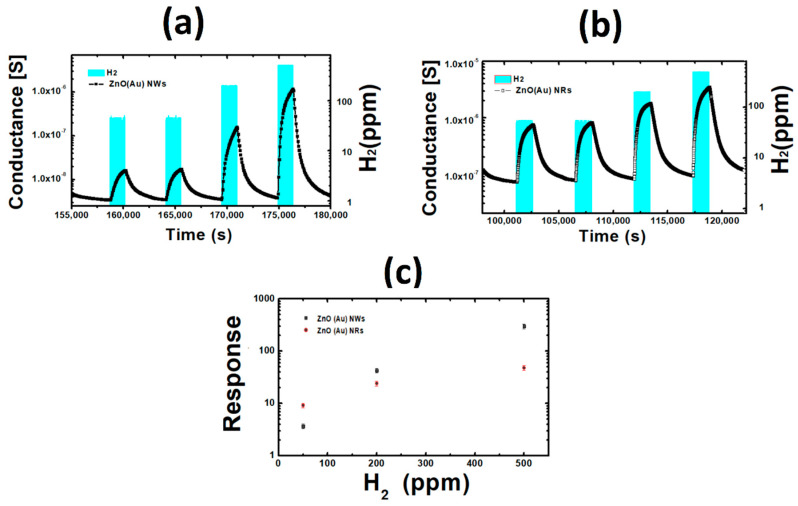
(**a**) Dynamic response ZnO NWs at 350 °C; (**b**) Dynamic response of ZnO NRs at 350 °C; (**c**) Calibration curves.

**Figure 10:**
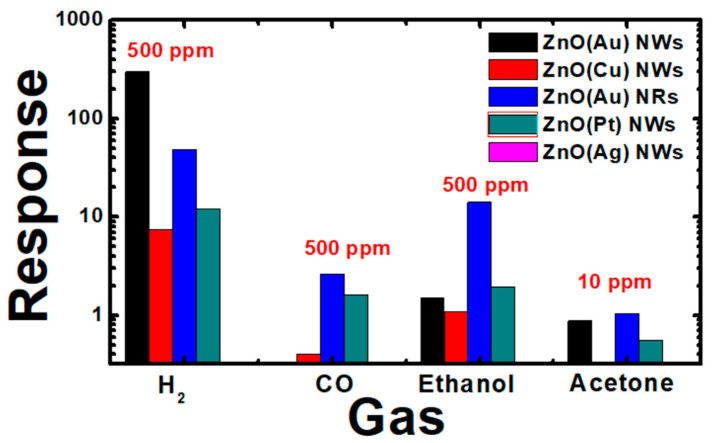
Selectivity histogram of 1D ZnO nanostructures using different catalysts.

Figure 9a,b reveal the dynamic response of ZnO (Au) nanowires and nanorods under exposure to 50, 200 and 500 ppm of H_2_. ZnO has an n-semiconducting nature, which explain the observed increase of the conductance. As reported in Figure 9c, ZnO response improves with the increase of hydrogen concentrations, and a high sensors response of about 300 for ZnO (Au) NWs and 50 for ZnO (Au) NRs was observed. Moreover, the signal is stable and recovering perfectly to the baseline level. These results are very interesting, compared to some studies reported in Table 4. This high response of ZnO (Au) NWs is attributed to the expected high specific area compared to bulk material and other morphologies. The sensors speed (sensors kinetic) was discussed by extracting the response/recovery time (Supplementary Materials Figure S1) from the prepared samples. The response time is defined as the time required by the sensor to reach the 90% of final conductance variation in presence of the gas. Conversely, the recovery time is the time needed for a sensor to reach 10% of conductance variation during the recovery. The response time was found to be similar for ZnO NWs and NRs (about 1200 s), while ZnO NWs showed a short recovery time of about 100 s. Furthermore, the capability of detection of low concentration of H_2_ (50 ppm) with stable baseline is verified. The selectivity of the sensors fabricated using all catalysts is displayed in Figure 10, showing that ZnO (Au) nanowires is a highly selective material toward H_2_ over CO, ethanol and acetone. It has been shown that ZnO decorated with Au shows high response towards H_2_ at high temperature (300 and 400 °C) while the sensing performance were poor at lower temperature which is maybe due to the high thermal energy that H_2_ needs to react with the pre-adsorbed oxygen through Au [37]. Instead, Pt decorated ZnO has shown good sensing capability toward H_2_ at 200 and 300 °C. These results are consistent with our work. Moreover, It has been reported previously that Ag oxidizes to Ag_2_O with p-type conductivity after an annealing treatment at 500 °C [38]. Therefore, the high response observed for ZnO (Ag) at high temperature (500 °C) may be attributed to the p-Ag_2_O/n-ZnO heterojunction formation at the interface. Similarly, p-CuO-n-ZnO heterojunction is formed in case of ZnO (Cu) sample, Cu catalyst was oxidized during the ZnO growth forming CuO. However, more elucidation is still needed to correlate the sensing performances with the nature of catalyst, since many factors such as the content (wt%) of catalyst on the surface (tip of the wire in our case), the shape, the aspect ratio and the possible oxidation of catalyst at high temperature should be taken into account [39].

## 4. Conclusions

This work reports the catalyst effect in the vapor liquid solid (VLS) growth of one-dimensional ZnO (1D ZnO) together with its effect on chemical sensors performances. The 1D ZnO nanostructures were successfully prepared using a low-cost method and catalyst (Au, Pt and Ag and Cu) supported growth following VLS mechanism as described in detail. The morphological, structural and electrical properties of the 1D nanostructures were studied. Depending on catalyst nature, different form, geometry, size and nanowires/nanorods abundance of ZnO were obtained. A morphology transition from nanowires to nanorods was observed using Au catalyst by increasing the deposition temperature and explained by coalescence effect of the Au catalyst seeds. ZnO crystallizes in hexagonal phase, while catalyst particles were shown in its metallic (Au, Pt and Ag) or oxidized (CuO) form. ZnO (Au) nanowires and nanorods, ZnO (Pt) nanowires and ZnO (Cu) nanowires polycrystalline structure have (002) as preferred orientation. Instead, for ZnO (Ag) nanowires both (010) and (002) are considered as preferential direction due to their almost identical *TC* values. The 1D ZnO nanostructures synthetized using different catalysts were tested under several reducing gases at several working temperatures: 200, 300, 350, 400 and 500 °C. ZnO (Au) nanowires and nanorods showed the best response to H_2_ at 350 °C. ZnO (Au) showed high response, good stability and selectivity to H_2_ with small response and recovery time, demonstrating their possible use for low-cost fabrication of high-performance chemical sensors. The signal was stable and recovered perfectly to the baseline level. Moreover, the ZnO (Au) nanowires was able to detect low H_2_ concentration (50 ppm). The response time was found to be similar for ZnO NWs and NRs (about 1200 s), while ZnO NWs showed a short recovery time (100 s). Selectivity has been observed towards hydrogen over other reducing gases. Most importantly, the effect of catalysts (Au, Pt, Ag and Cu) used in VLS for the growth on gas sensor mechanism was discussed. Finally, a possible explanation of the catalyst’s role in enhancing the conductometric sensors characteristics was presented, i.e., the chemical sensitization (spillover effect) induced by gold nanoparticle on the ZnO NWs tip.

## Figures and Tables

**Table 1:** Experimental parameters used in magnetron sputtering system for catalyst seed deposition.

Catalyst	Ar Flow (SCCM)	Pressure (10^−3^ mbar)	Magnetron Power (W)	Deposition Time (s)
Gold (Au)	7	5	75	5
Platinum (Pt)	7	5	75	2
Silver (Ag)	7	5	50	5
Cooper (Cu)	7	5	50	15

**Table 2:** The crystallite size and the average length of the 1D ZnO nanostructures using different catalysts.

Sample	Crystallite Size (nm)	Average ZnO Length (nm)
ZnO (Au) NWs	22	772 ± 47
ZnO (Au) NRs	22	442 ± 11
ZnO (Pt) NWs	30	840 ± 30
ZnO (Ag) NWs	31	342.1 ± 9.8
ZnO (Cu) NWs	24	≈4000

**Table 3:** The texture coefficient values of 1D ZnO nanostructures using different catalysts.

Sample	Diffraction Peaks		
	(010)	(002)	(011)
ZnO (Au) NWs	0.41	2.18	0.39
ZnO (Au) NRs	0.67	1.78	0.53
ZnO (Pt) NWs	0.80	1.53	-
ZnO (Ag) NWs	1.18	1.17	0.64
ZnO (Cu) NWs	0.75	1.67	0.57

**Table 4:** Studies reporting Hydrogen sensor based on ZnO nanomaterial.

Material	Technique	Temperature (°C)	Response/H_2_ (ppm)	Ref.
1D ZnO nano-assemblies	PE-CVD	400	13/5000	[40]
1D ZnO NWs	VLS process	400	90/300	[41]
ZnO Nanowires	Ultra-fast Microwave	250	0.95/500	[42]
Pd-decorated ZnO “nanosponge”	Supersonic cluster beam deposition (SCBD)	UV illumination, 20 °C	85/2%	[43]
ZnO nanobundles	nano-templating technique	350	20%/-	[44]
ZnO nanowires	electrochemical anodization	400	11.26/1000	[45]
Vanadium- doped ZnO thin film	Spray pyrolysis	300	55/500	[6]
ZnO two-dimensional nanostructures	thermal oxidation	175	5.37/200	[46]
Nanopillar ZnO	Two-step solution approach	350	28/2500	[47]
NPs-decorated networked ZnO NWs	Chemical vapor deposition (CVD)	Room temperature	4,6 (460%)/1000	[48]
ZnO NWs @ZIF-8	Vapor phase growth + Solvothermal	300	1.44/50	[49]
ZnO nanorods	facile one-pot galvanic-assisted technique	Room temperature	33/2000	[26]
p–n junction of ZnO thin films	D.C. sputtering technique + CVD	400	1.2/1000	[50]
ZnO thin films	Magnetron sputtering	350	98%/200	[51]
ZnO thin films	e-beam evaporation	400	59/40	[52]
Ni-doped ZnO thin film	RF sputtering	150	∼69%/10,000	[53]
Co:ZnO nanorods	hydrothermal method	150	53.7%/3000	[25]
ZnO nanowires	VLS	350	300 (30,000%)/500	This work

## Supplementary Materials

The following are available online at https://www.mdpi.com/2079-4991/10/10/1940/s1. Figure S1: The kinetic of ZnO (Au) NWs and ZnO (Au) NRs sensors. (a) Response and (b) recovery time of ZnO NWs based hydrogen sensor.
