# Supplementary material for: One Dimensional ZnO Nanostructures: Growth and Chemical Sensing Performances
Source: Nanomaterials (Basel). 2020 Sep 29;10(10):1940. doi: 10.3390/nano10101940 (PMC7599835; doi:10.3390/nano10101940)
Supplement: Supplementary file 1 [file nanomaterials-10-01940-s001.pdf]

Supplementary

One Dimensional ZnO Nanostructures: Growth and Chemical Sensing Performances

Abderrahim Moumen, Navpreet Kaur, Nicola Poli, Dario Zappa and Elisabetta Comini *

Sensor Lab, Department of Information Engineering, University of Brescia, Brescia 25123, Italy; a.moumen@unibs.it (A.M.); n.kaur001@unibs.it (N.K.); nicola.poli@unibs.it (N.P.); dario.zappa@unibs.it (D.Z.)

* Correspondence: elisabetta.comini@unibs.it; Tel.: +39-030-371-5877

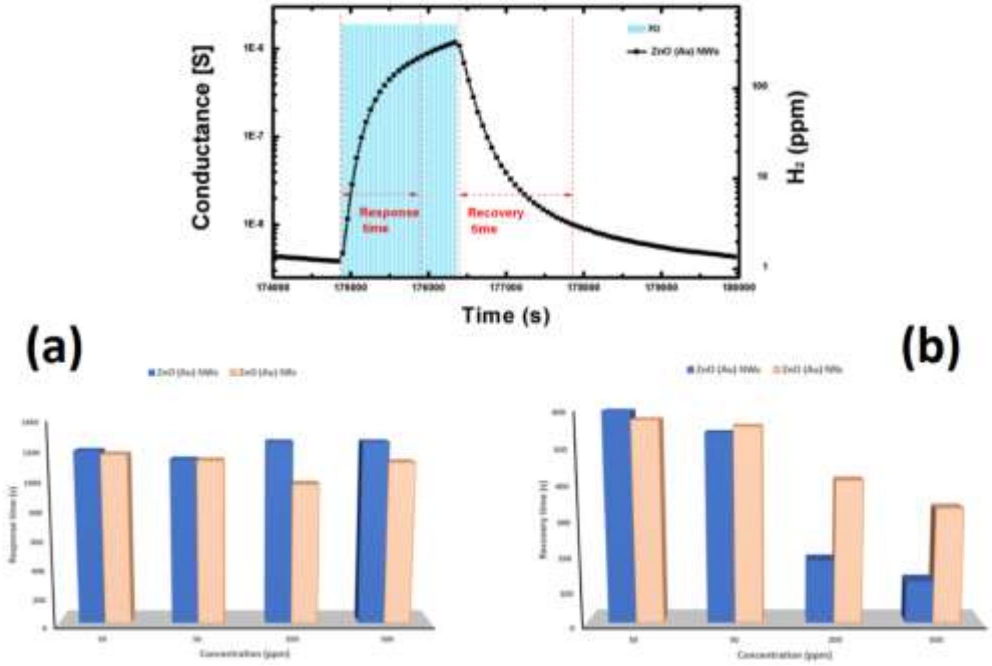

Figure S1. The kinetic of ZnO (Au) NWs and ZnO (Au) NRs sensors. (**a**) Response and (**b**) recovery time of ZnO NWs based hydrogen sensor.
